# Supplementary material for: Genetic and environmental influences on adult human height across birth cohorts from 1886 to 1994
Source: eLife. 2016 Dec 14;5:e20320. doi: 10.7554/eLife.20320 (PMC5156525; doi:10.7554/eLife.20320)
Supplement: Supplementary file 1. — (A) Height variance explained by additive genetic, shared environmental and unique environmental factors by birth year, sex and geographic-cultural region. (B) Model fit statistics for adult height by birth-year cohorts (all twin cohorts together). (C) Height variance and proportion of height variance explained by additive genetic, shared environmental and unique environmental factors by birth year, sex and geographic-cultural region. DOI: http://dx.doi.org/10.7554/eLife.20320.005 [file elife-20320-supp1.doc]

**Supplementary file 1A.** Height variance explained by additive genetic, shared environmental and unique environmental factors by birth year, sex and geographic-cultural region.

|  | Men |  |  |  |  |  |  |  |  |  |  |  | Women |  |  |  |  |  |  |  |  |  |  |
| --- | --- | --- | --- | --- | --- | --- | --- | --- | --- | --- | --- | --- | --- | --- | --- | --- | --- | --- | --- | --- | --- | --- | --- |
|  | Additive genetics | | |  | Shared environment | | |  | Unique environment | | |  | Additive genetics | | |  | Shared environment | | |  | Unique environment | | |
| Birth year | A | 95% CIs | |  | C | 95% CIs | |  | E | 95% CIs | |  | A | 95% CIs | |  | C | 95% CIs | |  | E | 95% CIs | |
| All cohorts |  |  |  |  |  |  |  |  |  |  |  |  |  |  |  |  |  |  |  |  |  |  |  |
| 1886-1909 | 30.2 | 26.6 | 32.4 |  | 0.0 | 0.0 | 3.1 |  | 8.5 | 7.7 | 9.4 |  | 19.1 | 15.6 | 22.7 |  | 4.7 | 2.8 | 7.7 |  | 12.4 | 11.4 | 13.5 |
| 1910-1919 | 35.7 | 33.1 | 38.3 |  | 1.7 | 0.0 | 4.3 |  | 6.0 | 5.6 | 6.4 |  | 22.9 | 20.4 | 25.5 |  | 3.8 | 2.9 | 6.2 |  | 7.4 | 6.9 | 8.0 |
| 1920-1929 | 31.5 | 29.9 | 33.1 |  | 6.0 | 4.4 | 7.6 |  | 6.1 | 5.8 | 6.3 |  | 23.9 | 22.0 | 26.0 |  | 4.2 | 3.2 | 6.1 |  | 6.0 | 5.7 | 6.4 |
| 1930-1939 | 29.4 | 27.2 | 31.8 |  | 4.1 | 2.6 | 6.3 |  | 6.6 | 6.2 | 7.0 |  | 25.2 | 23.5 | 27.0 |  | 3.9 | 2.9 | 5.6 |  | 5.2 | 4.9 | 5.5 |
| 1940-1949 | 30.9 | 29.3 | 32.7 |  | 5.6 | 5.3 | 7.3 |  | 5.3 | 5.1 | 5.5 |  | 27.8 | 26.5 | 29.2 |  | 3.2 | 2.5 | 4.5 |  | 4.6 | 4.4 | 4.8 |
| 1950-1959 | 32.0 | 30.3 | 33.6 |  | 7.1 | 5.4 | 8.7 |  | 5.1 | 4.9 | 5.4 |  | 28.1 | 26.9 | 29.5 |  | 5.8 | 4.4 | 7.1 |  | 4.8 | 4.6 | 5.0 |
| 1960-1969 | 33.4 | 30.6 | 36.4 |  | 7.5 | 5.6 | 10.4 |  | 7.2 | 6.8 | 7.6 |  | 29.6 | 27.7 | 31.6 |  | 5.8 | 5.6 | 7.8 |  | 6.8 | 6.5 | 7.1 |
| 1970-1979 | 37.4 | 34.7 | 40.3 |  | 5.5 | 2.7 | 8.3 |  | 5.3 | 5.1 | 5.6 |  | 29.9 | 28.0 | 31.9 |  | 8.3 | 7.8 | 10.3 |  | 5.5 | 5.3 | 5.7 |
| 1980-1994 | 41.7 | 38.0 | 45.2 |  | 2.6 | 0.0 | 6.3 |  | 5.3 | 5.0 | 5.7 |  | 28.4 | 26.1 | 30.9 |  | 9.2 | 6.7 | 11.6 |  | 5.4 | 5.2 | 5.7 |
|  |  |  |  |  |  |  |  |  |  |  |  |  |  |  |  |  |  |  |  |  |  |  |  |
| Europe |  |  |  |  |  |  |  |  |  |  |  |  |  |  |  |  |  |  |  |  |  |  |  |
| 1886-1909 | 29.8 | 26.4 | 32.1 |  | 0.0 | 0.0 | 3.0 |  | 8.6 | 7.8 | 9.5 |  | 18.1 | 14.4 | 21.8 |  | 5.2 | 3.1 | 8.2 |  | 12.8 | 11.7 | 14.0 |
| 1910-1919 | 33.8 | 31.8 | 35.8 |  | 0.0 | 0.0 | 2.6 |  | 5.8 | 5.2 | 6.4 |  | 22.0 | 19.2 | 24.8 |  | 3.3 | 1.9 | 5.8 |  | 8.0 | 7.3 | 8.7 |
| 1920-1929 | 26.9 | 24.2 | 29.7 |  | 5.4 | 4.6 | 8.0 |  | 6.8 | 6.2 | 7.4 |  | 23.5 | 21.3 | 25.8 |  | 3.1 | 2.7 | 5.1 |  | 6.2 | 5.8 | 6.7 |
| 1930-1939 | 29.1 | 26.8 | 31.6 |  | 4.2 | 2.9 | 6.5 |  | 5.6 | 5.2 | 6.1 |  | 24.5 | 22.6 | 26.4 |  | 2.8 | 1.5 | 4.7 |  | 5.1 | 4.8 | 5.4 |
| 1940-1949 | 30.5 | 28.7 | 32.4 |  | 4.1 | 3.4 | 6.0 |  | 5.0 | 4.7 | 5.3 |  | 25.9 | 24.5 | 27.3 |  | 2.8 | 2.4 | 4.2 |  | 4.2 | 4.0 | 4.4 |
| 1950-1959 | 30.1 | 28.3 | 32.1 |  | 6.7 | 4.8 | 8.6 |  | 5.0 | 4.8 | 5.3 |  | 27.4 | 25.9 | 28.9 |  | 3.2 | 2.4 | 4.7 |  | 4.2 | 4.0 | 4.4 |
| 1960-1969 | 34.0 | 30.4 | 38.0 |  | 6.8 | 4.8 | 10.5 |  | 5.2 | 4.8 | 5.6 |  | 29.7 | 27.2 | 32.4 |  | 3.2 | 1.5 | 5.7 |  | 5.1 | 4.8 | 5.4 |
| 1970-1979 | 37.5 | 34.5 | 40.7 |  | 4.4 | 2.4 | 7.4 |  | 4.5 | 4.3 | 4.8 |  | 29.5 | 27.2 | 32.0 |  | 5.9 | 4.3 | 8.3 |  | 4.3 | 4.0 | 4.5 |
| 1980-1994 | 40.8 | 36.2 | 44.6 |  | 2.0 | 0.0 | 6.7 |  | 4.2 | 3.9 | 4.6 |  | 26.2 | 23.5 | 29.2 |  | 10.5 | 7.4 | 13.4 |  | 4.1 | 3.9 | 4.4 |
|  |  |  |  |  |  |  |  |  |  |  |  |  |  |  |  |  |  |  |  |  |  |  |  |
| North America and Australia |  |  |  |  |  |  |  |  |  |  |  |  |  |  |  |  |  |  |  |  |  |  |  |
| 1886-1909 | 34.8 | 14.0 | 47.0 |  | 0.4 | 0.0 | 23.0 |  | 6.7 | 4.6 | 10.5 |  | 29.3 | 16.6 | 36.1 |  | 0.0 | 0.0 | 12.3 |  | 9.2 | 7.1 | 12.1 |
| 1910-1919 | 36.6 | 32.9 | 40.6 |  | 4.1 | 1.3 | 7.9 |  | 6.2 | 5.7 | 6.7 |  | 22.3 | 16.6 | 29.3 |  | 9.0 | 2.9 | 15.1 |  | 6.1 | 5.3 | 7.1 |
| 1920-1929 | 33.2 | 31.3 | 35.2 |  | 6.4 | 4.4 | 8.3 |  | 5.9 | 5.6 | 6.2 |  | 23.7 | 19.5 | 28.4 |  | 8.2 | 6.8 | 12.5 |  | 5.7 | 5.2 | 6.4 |
| 1930-1939 | 35.6 | 29.2 | 38.5 |  | 0.0 | 0.0 | 6.2 |  | 8.6 | 7.6 | 9.7 |  | 28.6 | 24.1 | 33.7 |  | 6.9 | 3.2 | 11.5 |  | 5.5 | 5.0 | 6.1 |
| 1940-1949 | 32.9 | 29.2 | 37.0 |  | 9.0 | 7.1 | 12.9 |  | 6.0 | 5.6 | 6.5 |  | 33.9 | 30.8 | 37.2 |  | 3.3 | 0.3 | 6.4 |  | 5.4 | 5.1 | 5.8 |
| 1950-1959 | 36.4 | 33.2 | 39.8 |  | 6.9 | 6.0 | 10.1 |  | 5.4 | 5.0 | 5.7 |  | 30.3 | 27.9 | 32.9 |  | 9.3 | 9.0 | 11.9 |  | 5.7 | 5.4 | 6.0 |
| 1960-1969 | 33.8 | 29.3 | 38.7 |  | 7.9 | 7.5 | 12.4 |  | 9.2 | 8.5 | 9.9 |  | 29.9 | 26.9 | 33.2 |  | 8.6 | 7.4 | 11.7 |  | 8.7 | 8.2 | 9.2 |
| 1970-1979 | 37.8 | 31.8 | 44.8 |  | 9.8 | 4.8 | 16.1 |  | 7.8 | 7.1 | 8.5 |  | 31.2 | 27.6 | 35.1 |  | 13.0 | 11.6 | 16.7 |  | 7.9 | 7.5 | 8.5 |
| 1980-1994 | 45.6 | 39.2 | 50.4 |  | 2.3 | 0.0 | 8.7 |  | 6.7 | 6.1 | 7.3 |  | 34.1 | 29.7 | 39.0 |  | 6.7 | 2.3 | 11.2 |  | 7.2 | 6.8 | 7.7 |
|  |  |  |  |  |  |  |  |  |  |  |  |  |  |  |  |  |  |  |  |  |  |  |  |
| East Asia |  |  |  |  |  |  |  |  |  |  |  |  |  |  |  |  |  |  |  |  |  |  |  |
| 1940-1949 | 35.3 | 14.9 | 60.6 |  | 5.0 | 0.0 | 34.0 |  | 2.3 | 1.3 | 4.8 |  | 19.5 | 4.9 | 35.7 |  | 5.3 | 0.0 | 25.4 |  | 2.8 | 1.8 | 4.7 |
| 1950-1959 | 13.8 | 6.0 | 24.5 |  | 4.9 | 0.0 | 15.9 |  | 2.7 | 1.9 | 4.0 |  | 10.8 | 4.0 | 25.5 |  | 12.1 | 0.0 | 22.6 |  | 2.6 | 2.0 | 3.6 |
| 1960-1969 | 24.7 | 13.3 | 39.8 |  | 9.0 | 0.0 | 22.8 |  | 3.1 | 2.4 | 4.1 |  | 23.3 | 16.9 | 27.9 |  | 0.0 | 0.0 | 6.7 |  | 2.0 | 1.6 | 2.5 |
| 1970-1979 | 27.3 | 16.6 | 35.4 |  | 2.7 | 0.0 | 14.7 |  | 2.2 | 1.8 | 2.7 |  | 24.1 | 16.1 | 32.6 |  | 5.1 | 0.0 | 14.0 |  | 1.5 | 1.3 | 1.8 |
| 1980-1994 | 35.4 | 20.7 | 43.8 |  | 0.0 | 0.0 | 15.8 |  | 5.0 | 3.8 | 6.7 |  | 17.2 | 10.0 | 28.0 |  | 9.0 | 0.0 | 17.3 |  | 3.2 | 2.7 | 3.8 |

**Supplementary file 1B.** Model fit statistics for adult height by birth-year cohorts (all twin cohorts together).

|  | Saturated model | | Full ACE sex-limitation | | Same parameters  for boys and girls | | Scale model  for sex | | No sex-specific  genetic effects | | AE model | |
| --- | --- | --- | --- | --- | --- | --- | --- | --- | --- | --- | --- | --- |
| Birth year | -2LL | d.f. | Δ-2LL^1^ | p-value^1^ | Δ-2LL^2^ | p-value^2^ | Δ-2LL^3^ | p-value^3^ | Δ-2LL^4^ | p-value^4^ | Δ-2LL^5^ | p-value^5^ |
| 1886-1909 | 57438 | 9145 | 18.8 | 0.1735 | 36.9 | <0.0001 | 36.9 | <0.0001 | 3.4 | 0.0664 | 9.2 | 0.0102 |
| 1910-1919 | 101377 | 16315 | 30.8 | 0.0059 | 119.9 | <0.0001 | 68.7 | <0.0001 | 0.7 | 0.4028 | 11.3 | 0.0035 |
| 1920-1929 | 211291 | 34097 | 29.5 | 0.0089 | 170.2 | <0.0001 | 35.4 | <0.0001 | 8.1 | 0.0044 | 68.1 | <0.0001 |
| 1930-1939 | 163774 | 26613 | 26.9 | 0.0198 | 105.5 | <0.0001 | 3.1 | 0.0783 | 8.5 | 0.0036 | 28.7 | <0.0001 |
| 1940-1949 | 317024 | 51479 | 13.4 | 0.4953 | 165.5 | <0.0001 | 2.9 | 0.0886 | 4.6 | 0.0320 | 59.3 | <0.0001 |
| 1950-1959 | 345065 | 55785 | 16.7 | 0.2725 | 108.6 | <0.0001 | 4.4 | 0.0359 | 21.0 | 0.0000 | 127.9 | <0.0001 |
| 1960-1969 | 211993 | 33661 | 27.5 | 0.0166 | 61.3 | <0.0001 | 3.8 | 0.0513 | 8.6 | 0.0034 | 53.1 | <0.0001 |
| 1970-1979 | 216915 | 34843 | 25.9 | 0.0267 | 45.9 | <0.0001 | 17.3 | <0.0001 | 1.8 | 0.1797 | 71.9 | <0.0001 |
| 1980-1994 | 153269 | 24617 | 22.6 | 0.0671 | 72.5 | <0.0001 | 25.8 | <0.0001 | 1.0 | 0.3173 | 47.5 | <0.0001 |

The fit of nested models was compared by calculating differences in -2 log-likelihood values (Δ-2LL), which follows the χ^2^-distribution with a difference in degrees of freedom (Δd.f.) that corresponds to the difference in the number of free parameters estimated.

^1^Compared to saturated model (Δd.f.=14); ^2^Compared to the full ACE sex-limitation model (Δd.f.=3); ^3^Compared to the full ACE sex-limitation model (Δd.f.=1); ^4^Compared to the full ACE sex-limitation model (Δd.f.=1); ^5^Compared to the full ACE sex-limitation model (Δd.f.=2).

**Supplementary file 1C.** Height variance and proportion of height variance explained by additive genetic, shared environmental and unique environmental factors by birth year and geographic-cultural region for men and women combined.

|  | Height variance | | | | | | | | | | |  | Proportion of height variance | | | | | | | | | | |
| --- | --- | --- | --- | --- | --- | --- | --- | --- | --- | --- | --- | --- | --- | --- | --- | --- | --- | --- | --- | --- | --- | --- | --- |
|  | Additive genetics | | |  | Shared environment | | |  | Unique environment | | |  | Additive genetics | | |  | Shared environment | | |  | Unique environment | | |
| Birth year | A | 95% CIs | |  | C | 95% CIs | |  | E | 95% CIs | |  | A | 95% CIs | |  | C | 95% CIs | |  | E | 95% CIs | |
| All cohorts |  |  |  |  |  |  |  |  |  |  |  |  |  |  |  |  |  |  |  |  |  |  |  |
| 1886-1909 | 23.6 | 21.0 | 26.3 |  | 2.8 | 0.0 | 5.1 |  | 10.7 | 10.1 | 11.5 |  | 0.64 | 0.57 | 0.71 |  | 0.07 | 0.01 | 0.14 |  | 0.29 | 0.27 | 0.31 |
| 1910-1919 | 30.8 | 29.0 | 32.7 |  | 1.9 | 0.0 | 3.6 |  | 6.6 | 6.3 | 6.9 |  | 0.78 | 0.74 | 0.83 |  | 0.05 | 0.00 | 0.09 |  | 0.17 | 0.16 | 0.18 |
| 1920-1929 | 29.6 | 28.4 | 30.8 |  | 4.9 | 3.7 | 6.0 |  | 6.0 | 5.9 | 6.3 |  | 0.73 | 0.70 | 0.76 |  | 0.12 | 0.09 | 0.15 |  | 0.15 | 0.14 | 0.15 |
| 1930-1939 | 28.6 | 27.4 | 29.9 |  | 2.5 | 0.0 | 3.7 |  | 5.7 | 5.5 | 6.0 |  | 0.78 | 0.74 | 0.81 |  | 0.07 | 0.04 | 0.10 |  | 0.16 | 0.15 | 0.16 |
| 1940-1949 | 30.2 | 29.3 | 31.1 |  | 3.3 | 2.4 | 4.2 |  | 4.9 | 4.7 | 5.0 |  | 0.79 | 0.76 | 0.81 |  | 0.09 | 0.06 | 0.11 |  | 0.13 | 0.12 | 0.13 |
| 1950-1959 | 31.3 | 30.5 | 32.2 |  | 4.8 | 3.9 | 5.7 |  | 4.9 | 4.8 | 5.1 |  | 0.76 | 0.74 | 0.78 |  | 0.12 | 0.10 | 0.14 |  | 0.12 | 0.12 | 0.12 |
| 1960-1969 | 32.9 | 31.5 | 34.3 |  | 4.8 | 3.4 | 6.1 |  | 6.9 | 6.7 | 7.2 |  | 0.74 | 0.71 | 0.77 |  | 0.11 | 0.08 | 0.14 |  | 0.16 | 0.15 | 0.16 |
| 1970-1979 | 34.3 | 33.0 | 35.7 |  | 6.0 | 4.6 | 7.3 |  | 5.4 | 5.3 | 5.6 |  | 0.75 | 0.72 | 0.78 |  | 0.13 | 0.10 | 0.16 |  | 0.12 | 0.11 | 0.12 |
| 1980-1994 | 37.4 | 35.8 | 39.2 |  | 2.9 | 0.0 | 4.6 |  | 5.4 | 5.2 | 5.6 |  | 0.82 | 0.78 | 0.86 |  | 0.06 | 0.03 | 0.10 |  | 0.12 | 0.11 | 0.12 |
|  |  |  |  |  |  |  |  |  |  |  |  |  |  |  |  |  |  |  |  |  |  |  |  |
| Europe |  |  |  |  |  |  |  |  |  |  |  |  |  |  |  |  |  |  |  |  |  |  |  |
| 1886-1909 | 23.1 | 20.4 | 25.9 |  | 2.9 | 0.0 | 5.2 |  | 11.0 | 10.2 | 11.8 |  | 0.63 | 0.55 | 0.70 |  | 0.08 | 0.01 | 0.14 |  | 0.30 | 0.28 | 0.32 |
| 1910-1919 | 27.6 | 25.4 | 29.7 |  | 1.3 | 0.0 | 3.3 |  | 7.0 | 6.5 | 7.5 |  | 0.77 | 0.71 | 0.82 |  | 0.04 | 0.00 | 0.09 |  | 0.19 | 0.18 | 0.21 |
| 1920-1929 | 25.5 | 23.8 | 27.2 |  | 3.7 | 0.0 | 5.3 |  | 6.5 | 6.1 | 6.8 |  | 0.72 | 0.67 | 0.76 |  | 0.10 | 0.06 | 0.15 |  | 0.18 | 0.17 | 0.19 |
| 1930-1939 | 28.1 | 26.8 | 29.5 |  | 1.9 | 0.0 | 3.2 |  | 5.3 | 5.1 | 5.6 |  | 0.79 | 0.76 | 0.83 |  | 0.06 | 0.02 | 0.09 |  | 0.15 | 0.14 | 0.16 |
| 1940-1949 | 28.5 | 27.6 | 29.5 |  | 2.9 | 2.0 | 3.9 |  | 4.5 | 4.3 | 4.7 |  | 0.79 | 0.77 | 0.82 |  | 0.08 | 0.06 | 0.11 |  | 0.13 | 0.12 | 0.13 |
| 1950-1959 | 29.8 | 28.8 | 30.9 |  | 3.6 | 2.6 | 4.6 |  | 4.5 | 4.4 | 4.7 |  | 0.79 | 0.76 | 0.81 |  | 0.10 | 0.07 | 0.12 |  | 0.12 | 0.11 | 0.12 |
| 1960-1969 | 32.8 | 31.0 | 34.6 |  | 3.3 | 0.0 | 5.0 |  | 5.1 | 4.9 | 5.4 |  | 0.80 | 0.75 | 0.84 |  | 0.08 | 0.04 | 0.12 |  | 0.12 | 0.12 | 0.13 |
| 1970-1979 | 33.5 | 32.0 | 35.0 |  | 5.0 | 3.4 | 6.5 |  | 4.4 | 4.2 | 4.6 |  | 0.78 | 0.75 | 0.82 |  | 0.12 | 0.08 | 0.15 |  | 0.10 | 0.10 | 0.11 |
| 1980-1994 | 35.5 | 33.5 | 37.6 |  | 3.7 | 0.0 | 5.8 |  | 4.2 | 4.0 | 4.4 |  | 0.82 | 0.77 | 0.87 |  | 0.09 | 0.04 | 0.13 |  | 0.10 | 0.09 | 0.10 |
|  |  |  |  |  |  |  |  |  |  |  |  |  |  |  |  |  |  |  |  |  |  |  |  |
| North America and Australia |  |  |  |  |  |  |  |  |  |  |  |  |  |  |  |  |  |  |  |  |  |  |  |
| 1886-1909 | 28.7 | 18.9 | 36.5 |  | 2.3 | 0.0 | 12.2 |  | 8.4 | 6.8 | 10.7 |  | 0.73 | 0.48 | 0.83 |  | 0.06 | 0.00 | 0.29 |  | 0.21 | 0.17 | 0.28 |
| 1910-1919 | 35.5 | 32.4 | 38.8 |  | 3.0 | 0.0 | 6.1 |  | 6.2 | 5.8 | 6.6 |  | 0.80 | 0.73 | 0.87 |  | 0.07 | 0.00 | 0.13 |  | 0.14 | 0.13 | 0.15 |
| 1920-1929 | 32.7 | 31.0 | 34.5 |  | 5.7 | 4.0 | 7.4 |  | 5.9 | 5.6 | 6.1 |  | 0.74 | 0.70 | 0.78 |  | 0.13 | 0.09 | 0.17 |  | 0.13 | 0.13 | 0.14 |
| 1930-1939 | 32.3 | 29.2 | 35.6 |  | 3.4 | 0.0 | 6.4 |  | 6.7 | 6.2 | 7.3 |  | 0.76 | 0.69 | 0.84 |  | 0.08 | 0.00 | 0.15 |  | 0.16 | 0.15 | 0.17 |
| 1940-1949 | 35.4 | 33.5 | 37.5 |  | 3.8 | 0.0 | 5.8 |  | 5.7 | 5.4 | 6.0 |  | 0.79 | 0.75 | 0.83 |  | 0.08 | 0.04 | 0.13 |  | 0.13 | 0.12 | 0.13 |
| 1950-1959 | 34.6 | 33.0 | 36.3 |  | 6.6 | 4.9 | 8.3 |  | 5.6 | 5.3 | 5.8 |  | 0.74 | 0.71 | 0.78 |  | 0.14 | 0.10 | 0.18 |  | 0.12 | 0.11 | 0.12 |
| 1960-1969 | 33.5 | 31.3 | 35.7 |  | 6.3 | 4.2 | 8.5 |  | 8.8 | 8.5 | 9.3 |  | 0.69 | 0.64 | 0.73 |  | 0.13 | 0.09 | 0.17 |  | 0.18 | 0.17 | 0.19 |
| 1970-1979 | 36.7 | 33.9 | 39.6 |  | 8.9 | 5.9 | 11.7 |  | 7.9 | 7.5 | 8.3 |  | 0.69 | 0.63 | 0.74 |  | 0.17 | 0.11 | 0.22 |  | 0.15 | 0.14 | 0.16 |
| 1980-1994 | 41.8 | 38.8 | 44.8 |  | 1.9 | 0.0 | 4.9 |  | 7.0 | 6.7 | 7.4 |  | 0.82 | 0.77 | 0.87 |  | 0.04 | 0.00 | 0.10 |  | 0.14 | 0.13 | 0.15 |
|  |  |  |  |  |  |  |  |  |  |  |  |  |  |  |  |  |  |  |  |  |  |  |  |
| East Asia |  |  |  |  |  |  |  |  |  |  |  |  |  |  |  |  |  |  |  |  |  |  |  |
| 1940-1949 | 25.6 | 14.6 | 40.3 |  | 6.0 | 0.0 | 20.4 |  | 2.6 | 1.8 | 4.0 |  | 0.75 | 0.42 | 0.95 |  | 0.18 | 0.00 | 0.51 |  | 0.08 | 0.05 | 0.12 |
| 1950-1959 | 16.0 | 9.0 | 24.5 |  | 5.1 | 0.0 | 13.4 |  | 2.7 | 2.1 | 3.4 |  | 0.67 | 0.37 | 0.91 |  | 0.21 | 0.00 | 0.52 |  | 0.11 | 0.08 | 0.15 |
| 1960-1969 | 28.1 | 24.5 | 32.3 |  | 0.0 | 0.0 | 3.9 |  | 2.4 | 2.1 | 2.9 |  | 0.92 | 0.80 | 0.94 |  | 0.00 | 0.00 | 0.12 |  | 0.08 | 0.06 | 0.10 |
| 1970-1979 | 29.6 | 26.6 | 33.0 |  | 0.0 | 0.0 | 6.4 |  | 1.7 | 1.5 | 2.0 |  | 0.94 | 0.93 | 0.95 |  | 0.00 | 0.00 | 0.20 |  | 0.06 | 0.05 | 0.07 |
| 1980-1994 | 30.1 | 25.5 | 34.2 |  | 0.0 | 0.0 | 4.3 |  | 3.8 | 3.2 | 4.4 |  | 0.89 | 0.77 | 0.91 |  | 0.00 | 0.00 | 0.12 |  | 0.11 | 0.09 | 0.13 |
